# SATB1-mediated chromatin landscape in T cells

**DOI:** 10.1080/19491034.2020.1775037

**Published:** 2020-06-10

**Authors:** Tomas Zelenka, Charalampos Spilianakis

**Affiliations:** a Department of Biology, University of Crete, Heraklion, Crete, Greece; b Gene Regulation & Genomics, Institute of Molecular Biology and Biotechnology—Foundation for Research and Technology Hellas, Heraklion, Crete, Greece

**Keywords:** SATB1, CD4 cells, thymocytes, nuclear matrix, phase separation, chromatin organization, T cell receptor, autoimmunity, cancer

## Abstract

The regulatory circuits that define developmental decisions of thymocytes are still incompletely resolved. SATB1 protein is predominantly expressed at the CD4^+^CD8^+^cell stage exerting its broad transcription regulation potential with both activatory and repressive roles. A series of post-translational modifications and the presence of potential SATB1 protein isoforms indicate the complexity of its regulatory potential. The most apparent mechanism of its involvement in gene expression regulation is via the orchestration of long-range chromatin loops between genes and their regulatory elements. Multiple SATB1 perturbations in mice uncovered a link to autoimmune diseases while clinical investigations on cancer research uncovered that SATB1 has a promoting role in several types of cancer and can be used as a prognostic biomarker. SATB1 is a multivalent tissue-specific factor with a broad and yet undetermined regulatory potential. Future investigations on this protein could further uncover T cell-specific regulatory pathways and link them to (patho)physiology.

## Introduction

The adaptive immune response is fully dependent on the proper T cell development in the thymus whose major stages can be characterized by the expression of CD4 and CD8 glycoproteins. The early T cell progenitors do not express either of them, hence they are referred as double negative (DN) T cells [1]. Precursors of αβ T cells at the DN3 stage undergo β selection, requiring signaling via a pre-TCR (T Cell Receptor) consisting of a properly rearranged TCRβ chain, CD3 chains and the pre-Tα [1–3]. These cells start expressing CD4 and CD8 surface markers and transition into CD4^+^CD8^+^ double positive (DP) cells. In the T cell lineage, the process called positive selection ensures the maturation of T cells whose receptors recognize self major histocompatibility complex molecules. This facilitates the survival of lymphocytes, a developmental event that is linked to lineage commitment, the process by which lymphocyte subsets are generated. On the contrary, those that are selected undergo a series of maturational changes while migrating from the thymic cortex to the medulla. There, they are further challenged via the process of negative selection where they are exposed to self-antigens [1,4]. All the aforementioned events are tightly coordinated by a number of transcription factors. They target stage-specific genes and contribute to shaping the nuclear architecture via epigenetic modifications and overall chromatin organization, which are all ultimately linked to changes in gene expression. The Special AT-rich Binding protein 1 (SATB1) is a T cell-specific factor involved in both the epigenome as well as the 3D chromatin organization [5–9]; however, a complete understanding of all its effector roles still remains elusive. Recently, SATB1 has been studied in multiple clinico-pathological settings, especially cancer-related – as reviewed by several groups [10–12]. In this review, we focus on the physiological role of SATB1 during T cell development with respect to its direct cell-intrinsic implication and suggest new directions in SATB1 research.

## General aspects of SATB1 structure & function

The *Satb1* gene is located on chromosome 17 in mouse and on chromosome 3 in humans. It encodes for several transcript isoforms utilizing multiple promoters whose selection in T cells is NF-κB signaling-dependent [13]. According to the Ensembl genome database (ENSMUSG00000023927), murine SATB1 is present in two protein isoforms being 764 and 795 amino acids long. The mRNA coding human long SATB1 isoform is deposited in the GenBank database (accession number AB209761), however the presence of the long isoform in any organism has not been experimentally validated so far. Commercially available antibodies cannot discriminate between the two isoforms, yet all the published conclusions are arguably made about the short isoform, including all the heterologous expression systems. Although, human and mouse SATB1 proteins share 98.3% identity (13 amino acids difference), in this review, we mainly focus on murine SATB1.

**Figure 1.**
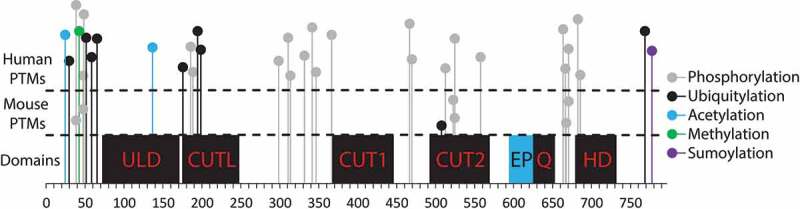
Structure of SATB1 protein and its potential post-translational modifications. Along the x-axis representing the amino acid positions in SATB1 protein, are depicted its important structural features and domains. ULD – ubiquitin-like domain, CUTL – CUT-like domain, CUT1 and CUT2 domains, EP – the peptide encoded by the predicted extra exon of the long SATB1 isoform, Q – compositional bias represented by a poly-Q domain and a stretch of prolines, HD – homeodomain. In the two upper segments separated by the dashed lines, there are potential murine and human post-translational modifications, extracted from the PhosphoSitePlus® database [44]. The abundance of gray lollipop visualizations in the chart indicate a number of sites that could potentially be phosphorylated and thus hold a great potential to regulate valency of SATB1 and affect its biophysical and/or regulatory properties.

A nuclear localization signal is present at the N-terminus [residues 20–40; 14], followed by an oligomerization domain which is similar in sequence to the PDZ domain [15]. Structurally, the oligomerization domain rather resembles ubiquitin – hence it is alternatively called ULD (ubiquitin-like domain, Figure 1; 16). Dimerization of the N-terminal domain is required for DNA binding [17] and further tetramerization of these dimers is a proposed mechanism mediating chromatin loops [16,18]. The N-terminal domain itself can interact with chromatin, likely due to the presence of the CUT-like domain [16,18–20], however other domains are necessary for the stabilization of this interaction [21]. Namely, there are two CUT domains and a homeodomain (Figure 1; 22), all contributing to DNA binding. The CUT domains determine affinity and the homeodomain ensures specificity [21]. Several independent studies revealed a DNA binding motif with prominent TAATA sequence [17,20,21] which corresponds to the original characterization of SATB1 as a Special AT-rich Binding protein [23]. However, SATB1 binds only a very small fraction of the TAATA motifs available in the genome, hence the current models calculate with different determinants of chromatin targeting such as SATB1’s preferential binding to nucleosome-dense regions and favoring sites with negative torsional stress [21].

**Figure 2.**
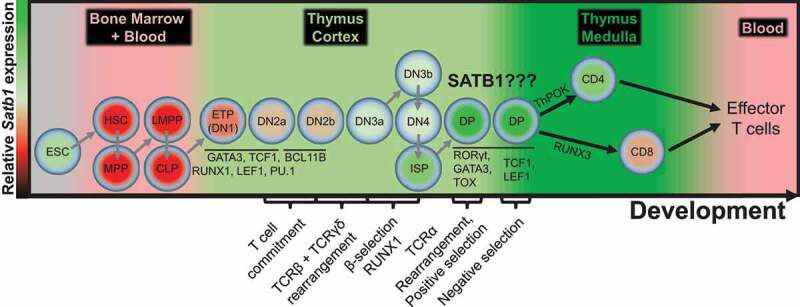
*Satb1* expression in relation to early T cell development. Color of depicted cell types indicates relative *Satb1* gene expression. The background color symbolizes distinct body compartments harboring the depicted cell types. *Satb1* is predominantly expressed during the double positive stage of T cell development, indicating a potential role of SATB1 at this stage. Important master regulators and key events of the T cell development are also depicted to better understand the processes and the genes which SATB1 could regulate. ESC – embryonic stem cell, HSC – hematopoietic stem cell, MPP – multipotent precursor, LMPP – lymphoid-primed multipotent precursor, CLP – common lymphoid precursor ‘A’ type, ETP – early T cell precursor, DN – CD4CD8 double negative T cell, ISP – immature single-positive T cell, DP – CD4CD8 double positive T cell, CD4 – CD4 single positive T cell, CD8 – CD8 single positive T cell.

*Satb1* is not ubiquitously expressed, however its importance in many, yet unrecognized tissues, is gradually gaining attention. So far, SATB1 was largely studied in embryogenesis [24–27] and neurogenesis [28,29] but it also employs certain roles during skin development [30], in ameloblasts [31] and in the liver [32]. Nevertheless, SATB1 research is mostly focused on hematopoiesis [33–37], specifically in the lymphoid lineage, even though SATB1 may also play certain roles during erythropoiesis [27,38]. This is partially due to the highest *Satb1* expression during T cell development [37], mainly at the CD4^+^CD8^+^ double positive and immature CD4SP stages (Figure 2; 39, 40, 41). There is not much known about the transcriptional regulation of *Satb1* in the thymus. In thymocytes, *Satb1* is positively regulated by TCR signaling and directly by GATA3 binding to its proximal regulatory elements [42]. During Th2 cell differentiation it is positively regulated by IL4 and NF-κB signaling [13]. In regulatory T cells, *Satb1* is negatively regulated by a two-layer system; it is directly repressed by FOXP3 and also post-transcriptionally by FOXP3-induced microRNAs miR-155, miR-21, miR-7, miR-34a, and miR-18a [43].

The function of SATB1 at the protein level is regulated by post-translational modifications. A summary of post-translational SATB1 modifications based on the PhosphoSitePlus® database [44] is depicted in Figure 1. SATB1 can be phosphorylated at serine and threonine residues [45]. Phosphorylation of SATB1 at S185 by PKC increases its DNA binding activity [45]. Moreover, phosphorylation at the N-terminal part of the protein leads to preferential binding to histone deacetylase HDAC1 [45]. SATB1-mediated recruitment of HDAC1 results in repression of the *Il5* gene expression [46]. Conversely, dephosphorylated SATB1 interacts with p300/CBP-associated factor (PCAF) which then acetylates SATB1 at lysine K136. This consequently leads to decreased DNA binding by SATB1 and possibly upregulation of gene expression [45]. SATB1 also interacts with p300/CBP [45] which indicates its involvement in positive T cell chromatin regulation, as shown in K562 cells [38]. Moreover, phosphorylation also regulates sumoylation of SATB1, promoting its cleavage by caspase 6 in PML bodies [15,47,48]. Most of the published experiments studying the post-translational modifications of SATB1 are focused on the N-terminal part of the protein. Modifications in this region can potentially affect both the oligomerization of SATB1 molecules as well as its interaction with chromatin modifying enzymes, transcription factors and other partners. It is not clear yet, what would be the role of post-translational modifications at the C-terminus where DNA-binding domains are located together with the poly-Q domain and other elements (Figure 1). However, such modifications could serve as another switch, regulating DNA binding properties and consequently formation of the SATB1-mediated chromatin loops.

## Biological impact of SATB1 deletion in T cell development

To understand the regulatory functions of SATB1 during T cell development, a series of *Satb1* mutated mice have been developed. Whole-body *Satb1*^–/ –^ knockout animals die within a few weeks upon birth, likely due to severe developmental problems [30,37,49]. These animals evince a small thymus consisting mainly from the cortex with sparse medulla [49]. There is a blockade at the DP stage of T cell development, resulting in increased numbers of DP thymocytes and fewer CD4/CD8 single positive (CD4SP/CD8SP) cells [49]. SATB1 may already play certain roles during lymphoid lineage commitment, as shown by decreased numbers of early T cell precursors in *Satb1*^fl/fl^*Vav-*Cre^+^ mice [SATB1 removal from all hematopoietic cells; 40], which is less manifested in the *Satb1*^fl/fl^*Lck-*Cre^+^ animals [SATB1 removal after the DN2 stage; 40]. Such discrepancy indicates that SATB1 plays a certain role already in pre-thymic stages, which was already validated by several studies [33–37]. However, *Satb1* is predominantly expressed during the DP stage (Figure 2; 39, 40, 37, 41), indicating its importance there. To study the potential roles of SATB1 at this stage, *Satb1*^fl/fl^*Cd4*-Cre^+^ conditional knockout animals have been created [SATB1 deletion induced at the DP stage; 50, 39]. Similarly to the other knockouts, these animals have increased numbers of DP cells indicating an alteration in positive selection. This was further confirmed by crossing SATB1-deficient animals with OT-I^+^ and OT-II^+^ mice. The resulting animals have thymocytes expressing ﬁxed TCRs which, upon positive selection, should give rise to CD8^+^ and CD4^+^ cells, respectively. Both OT-I^+^ and OT-II^+^ in SATB1-deficient background gave rise to less than expected CD8SP and CD4SP cells, respectively, indicating a developmental blockade [40]. Moreover, about 50% of OT-II^+^ thymocytes in SATB1-deficient background developed into CD8SP T cells, suggesting the importance of SATB1 in T cell lineage specification [50]. The aforementioned *Satb1* knockout animals develop autoimmune and inflammatory phenotypes. This was partially explained by a deficiency in regulatory T cells [39,40]. Notably, other knockout animals deleting SATB1 downstream of the DP stage, namely *Satb1*^fl/fl^*Thpok*-Cre^+^ and *Satb1*^fl/fl^*Foxp3*-Cre^+^, do not manifest autoimmunity and even the CD4SP and CD8SP cell populations do not display any major changes [39,41]. Collectively, these findings underpin the importance of SATB1 during the DP stage of T cell development, although the detailed molecular mechanisms of its mode of action are still missing.

## Regulation of gene expression in T cells

Transcriptional programs in early T cell progenitors (DN1 and DN2a) are regulated by two modes of function of the transcription factor PU.1 (encoded by *Spi1*). PU.1 exerts its action not only by interacting and recruiting SATB1 and RUNX1 to target genes but also by titrating out the same partners for binding to their targets thus leading to gene repression [51]. A study of early developing T cells utilizing methods targeting chromatin organization and its accessibility demonstrated that there are two events of abrupt genome-wide changes – during T cell lineage commitment from DN2 to DN3 and during the transition from DN4 to DP stage [52]. T cell lineage commitment during the transition from DN2 to DN3 is coordinated by the factor BCL11B [53–55] together with TCF1 which is responsible for setting up the epigenetic landscape [56,57] and likely by other factors [58,59]. Moreover, TCF1 is important in the second wave of chromatin rearrangements during the transition from DN4 thymocytes to DP and SP stages [52,57]. This is also a point when *Satb1* starts being highly expressed [37,39–41] and a point when the *Satb1*^fl/fl^*Cd4*-Cre^+^ animals abort its production. The accessibility of chromatin regions inversely correlates with nucleosome density. In fact, nucleosome positioning itself is a cell type-specific feature of differentiating T cells [60]. SATB1 binds DNA sequences embedded in nucleosomal cores [21] and it is known to recruit the chromatin remodeling enzymes ACF and ISWI [9]. Therefore, SATB1 binding in cooperation with chromatin remodeling enzymes can be enough to shape the T cell chromatin landscape. Moreover, in CD8^+^ T cells, SATB1 recruits the nucleosome remodeling deacetylase (NuRD) repressive complex to regulatory elements of the *Pdcd1* gene locus (encoding PD1) to suppress its expression [61]. Therefore, in T cell specific *Satb1* deficient mice, PD1 is expressed at high levels and tumor immunity is impaired. The sustained expression of PD1 is linked to chronic infections and cancer due to CD8 cell exhaustion, hence SATB1 likely controls both PD1 expression and anti-tumor T cell responses [61].

Recently, the importance of higher order chromatin structure, especially interactions between genes and their regulatory elements is becoming highly appreciated as a mechanism of gene expression regulation. Therefore, next, we review SATB1 as a potential determinant of T cell chromatin architecture.

## T cell chromatin organization and the roles of SATB1

Chromatin is organized into several types of domains. The most frequently discussed units of chromatin organization are topologically associated domains (TADs), however the recent findings propose more appropriate terms such as contact, loop and compartmental domains [62–68]. Chromatin segmentation in mammals is often driven by architectural proteins such as CTCF and the cohesin complex [69–71]. Recent findings [66,72] support the idea that 3D organization is determined by the transcriptional state of chromatin [73], i.e. by the presence of the transcription machinery, transcription factors, epigenetic marks, chromatin accessibility, etc. All these factors could regulate the chromatin architecture through cohesin loading and CTCF binding in a tissue-specific manner during cell development and differentiation [74]. However, the question whether there could be a tissue-specific gene expression regulation through direct, protein-based, 3D chromatin organization, still remains posed. In contrast to ubiquitously expressed CTCF, YY1 and other architectural proteins, SATB1 is a good candidate for further research as a potential T cell-specific genome organizer.

The roles of traditional structural proteins in T cell chromatin organization and consequently gene expression regulation have been recently reviewed [75,76]. The first proof of long-range interactions in T cells was documented for the Th2 locus of naive CD4^+^ differentiating T cells where the promoters of the cytokine genes *Il4, Il5* and *Il13* were shown to be co-regulated by long-range interaction with the Th2 locus control region [77]. However, the final production of Th2-specific cytokines seems to be the result of a more complicated network of protein factors and long-range chromatin interactions. Namely, the *Il5* gene is repressed by direct SATB1 binding [46]. In contrast, SATB1-mediated recruitment of β-catenin and p300 positively regulates *Gata3* expression and thus also the levels of signature Th2 cytokine genes *Il4, Il10*, and *Il13* [78]. Moreover, the *Il4* gene is synergistically activated by JUNB, SATB1 and coactivators such as P300 and PCAF [79]. However, SATB1 also mediates multiple loops at the Th2 locus [80]. Therefore, an additional mechanism of positive regulation at the Th2 locus could involve SATB1-mediated chromatin loops connecting the locus with its enhancers. DNA affinity chromatography followed by mass spectrometry already confirmed SATB1 binding to RHS6 [*Rad50* Hypersensitive Site 6; 81], probably the most prominent DNase I hypersensitive site of the Th2 locus control region [82,83].

Currently, one of the most appreciated roles of SATB1 is attributed to the development of regulatory T cells. SATB1 establishes an enhancer network associated with Treg signature genes including the transcription factor FOXP3 [39]. FOXP3 itself can participate in chromatin organization of Tregs [84]; however, SATB1 operates earlier in the thymus – in Treg precursor cells [39]. SATB1 binds closed chromatin and triggers the Treg differentiation pathway through the induction of repressed chromatin [39] in an IL-2-dependent manner [85]. Interestingly, a mutation in FOXP3 leads to increased enhancer-promoter interactions at the Th2 locus and as a result, these mutated regulatory T cells exhibit Th2 effector function including expression of Th2 cytokines [86]. A similar observation for the development of Treg cells with Th2 properties was shown in *Msc*^−/−^ animals (lacking the transcription factor musculin) as a result of reduced *Foxp3* expression and derepressed GATA3 activity [87]. SATB1 is known to organize the Th2 locus [80] and it is also under FOXP3 regulation [43], hence it could also be the link between Treg and Th2 cell lineage programs in these studies.

The autoimmune phenotype of *Satb1*^fl/fl^*Cd4*-Cre^+^ animals is explained by the deficiency of Treg cells due to alteration of the regulatory network [39]. However, these knockout animals manifest overall decreased numbers of CD4SP and CD8SP cells [39,41], hence the observed autoimmune phenotype may be linked to deregulation of other pathways too. The hallmark of the DP stage of T cell development is to ensure the controlled production of CD4 and CD8 cells. SATB1 is directly involved in controlling the regulatory elements of crucial CD4 and CD8 lineage specifying genes, namely *Zbtb7b* (encoding ThPOK), *Runx3* and *Cd4* and *Cd8* co-receptors themselves [50]. SATB1 is detected on the enhancers of these genes, likely mediating their activation and/or enhancer-promoter communication during a time window after positive selection – but not in the later developmental stages [50]. This indicates that SATB1 plays indeed multiple roles in the chromatin organization of T cells. However, a genome-wide study probing the SATB1-mediated chromatin architecture in developing T cells is still missing.

The T cell receptor gene loci are commonly utilized as a model to study the effect of chromatin organization as an epigenetic determinant of gene regulation in physiological processes. The polypeptide chains that comprise the functional TCR are derived from the α and β (and/or γ and δ) TCR gene loci. These loci consist of multiple genomic segments of variable (Vα), joining (Jα), and constant (Cα) regions for the TCRα gene locus and Vβ, Dβ (diversity), Jβ, and Cβ gene segments for the TCRβ gene locus [88]. Creation of a functional TCR is fully dependent on somatic recombination of the aforementioned genomic segments, based upon the action of protein complexes that include RAG1 and RAG2 recombinases. The transcriptional activity of *Rag1* and *Rag2* genes is tightly regulated in the different stages of T cell development [89]. *Rag1* and *Rag2* gene expression is regulated via long-range chromatin interactions between the *Rag* gene promoters and distal regulatory elements from the *Rag2* gene that act as enhancer elements for the expression of recombinases in DP thymocytes [90]. This enhancer, or anti-silencer element counteracts the negative transcription regulation mediated by an intergenic silencer bound by RUNX transcription factors. SATB1 is responsible for mediating the long-range interaction between the *Rag2* gene and the anti-silencer element and for loading *Rag1* and *Rag2* promoters with RNA polymerase II. In SATB1-deficient thymocytes, *Rag1/2* genes are expressed in lower levels at the DP stage and this is associated with partially impaired *Tcra* gene rearrangements [91]. TCR gene loci undergo major conformational changes during the different stages of development with the ultimate goal to create large chromatin loops and bring distal DNA segments in close proximity to support recombination events. These essential chromatin arrangements are orchestrated by architectural protein CTCF and members of the cohesion complex as documented by multiple studies [92–94]. Following the impaired *Tcra* gene rearrangements in *Satb1* knockout mice [91], it would be interesting to investigate chromatin organization at the TCR locus of these animals as an additional factor to the lower level of RAG proteins, possibly contributing to the phenotype. Additionally, it would be appealing to unravel the potential functional interplay between ubiquitously expressed and cell-specific genome organizers.

## Past, current & future perspectives

### Nuclear matrix and SATB1

SATB1 was primarily considered as a protein of the nuclear matrix [23]. Nuclear matrix was understood as a ﬁlamentous network consisting mainly from architectural proteins (e.g. CTCF), RNA-binding proteins (e.g. heterogeneous nuclear ribonucleoproteins; hnRNPs) and RNA, creating a scaffold for chromatin modifying enzymes and chromatin loops via the attachment to AT-rich DNA [95]. This concept has become outdated mainly due to the inability to reproduce the findings in living cells, indicating that nuclear matrix could be a cell treatment artifact [96,97]. Arguments against the nuclear matrix involve generation of a filamentous structure due to high salt treatment [96]. Nowadays, one of the major concepts of nuclear compartmentalization and consequent regulation of gene expression is a process called liquid-liquid phase separation (LLPS). Phase separating proteins have the ability to create biomolecular condensates, driven by weak, multivalent interactions between macromolecules [98–100]. Formation of biomolecular condensates is dependent on a number of factors including salt concentration, pH, temperature and often also RNA presence [100,101]. Changes of these variables can either prevent or promote phase separation; however, after passing a certain threshold the phase separating proteins may aggregate into fibrillar solid-like structures [102]. These aggregates are known from several pathological situations [103,104]. Experimental high salt treatment of aged droplets of protein Whi3 also resulted in elongated fibers [105] and similar solid-like structures of FUS were achieved by RNA removal [106]. Hence, we propose that the altered conditions during nuclear matrix preparation could induce aggregation of the phase separating proteins, producing an artificial meshwork of the nuclear matrix. This would imply that the proteins of nuclear matrix can undergo phase separation under certain conditions. Some proteins of nuclear matrix such as SAF-B [107] and hnRNPA1 [108] were already proven to phase separate and even create ordered solid phase fibrillar structures. Hence, in the next chapter, we speculate that phase separation could also represent another mode of transcriptional regulation by SATB1.

### A possible link between SATB1 and phase separation

**Figure 3.**
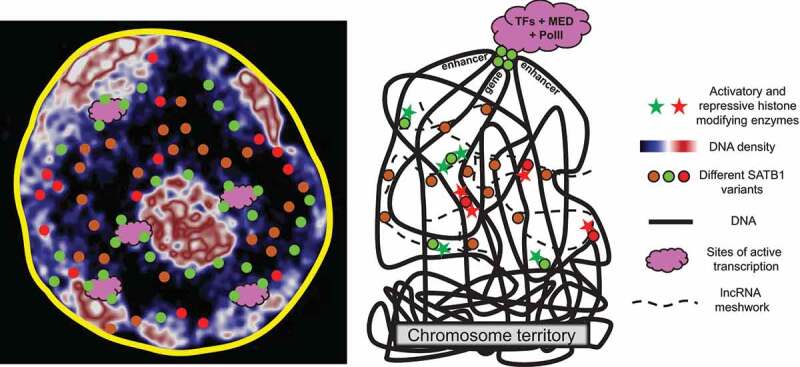
SATB1 in developing T cells mainly localizes to interchromatin regions and to the euchromatin/heterochromatin boundary. This localization pattern surrounding central heterochromatin, typical for T cells, was originally described as a cage-like structure. Super-resolution images indicate that the interconnected cage may instead correspond to individual scattered spheres and tendrils. Here we suggest that SATB1 exists in multiple variants such as the predicted isoforms of variable length (see Figure 1). Additionally, SATB1 is post-translationally modified including phosphorylation, methylation, sumoylation, yielding multiple SATB1 variants with potentially distinct functions. A hypothesis is that some SATB1 variants may be responsible for purely the structural organization of the nucleus (brown dots) in cooperation with the previously recognized elements of the nuclear matrix, such as the meshwork of lncRNAs. Other variants may have either repressive (red dots) or activatory (green dots) roles, depending on the chromatin modifying enzymes that SATB1 recruits (see Figure 5). On top of that, SATB1 possibly organizes the genome keeping certain regions looped out from heterochromatin. Another variant of SATB1 can further loop out genes and regulatory elements and bring them to transcriptional condensates via its IDR targeting or by other mechanisms to ensure another mode of positive transcriptional regulation.

The subcellular localization of SATB1 in thymocyte nuclei displays a cage-like pattern confined to interchromatin/low-DNA-content regions of nuclei (Figure 3; 5). A recent study employing super-resolution microscopy methods presented SATB1 as small spherical and tendril-like structures [21]. The phase separated transcriptional condensates appear as small spheres at the border of euchromatin and heterochromatin [109]. Condensates are generally excluded from bulk chromatin [110], all similar to localization of thymocyte SATB1 (Figure 3; 49, 21).

**Figure 4.**
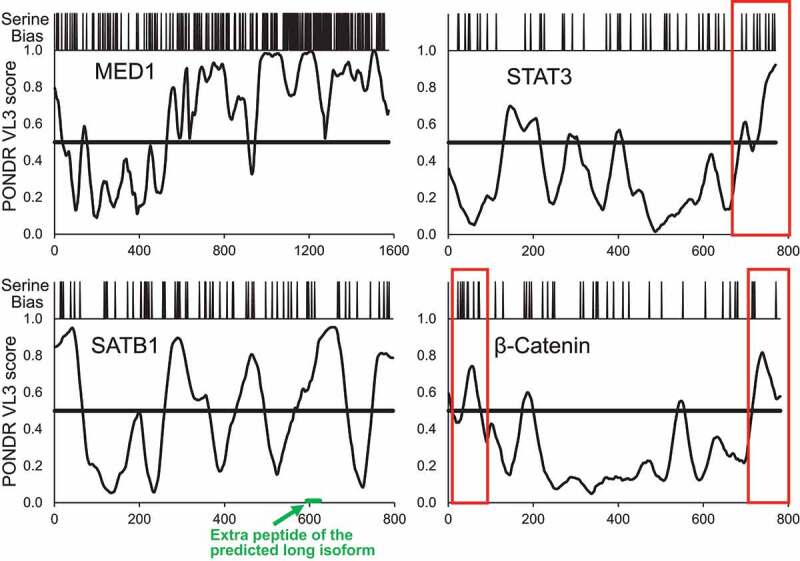
Prediction of intrinsically disordered regions (IDR) using VL3 PONDR score (http://www.pondr.com/). In order to understand the potential of SATB1 to be involved in phase separation, we indicate VL3 PONDR score of intrinsically disordered region and a serine bias above scores. The scores above the horizontal line indicate IDR. SATB1 is compared to MED1, a protein known to be involved in phase separation [141] and two signaling molecules STAT3 and β-Catenin which were shown to target genes into mediator condensates via their IDR regions – visualized by red rectangles [116]. SATB1 does not evince such a strong disordered region like MED1, however regions between its structural domains display comparable or even stronger IDRs than those of the signaling molecules STAT3 and β-Catenin. We propose a model in which SATB1 could regulate gene expression via 3D chromatin organization and targeting genes to the transcriptionally active condensates via its IDR. [x-axis: amino-acid position, y-axis: PONDR (prediction of natural disordered regions) score].

Typical phase separating proteins are capable of multivalent protein-protein interactions assembling high order oligomers or weak multivalent interactions between intrinsically disordered regions (IDRs) and often accompanied by interactions with RNA [98,100]. The multivalency can be regulated by various post-translational modifications [111,112]. For example, phosphorylation of RNA polymerase II regulates its incorporation into condensates associated with either transcription initiation or splicing [113]. The N-terminus of SATB1 is naturally multivalent as it promotes oligomerization [15,16,18] and protein-protein interactions with other factors and protein complexes [5,9,114,115]. Post-translational modifications and especially phosphorylation plays an important role in regulating the SATB1 function [45]. Based on predictions and mass spectrometry data, there are multiple sites on SATB1 that can be phosphorylated under certain conditions (Figure 1) and likely affect the function of SATB1. However, further studies and experimental validation of these potential SATB1 variants are needed. Here we speculate, that these modifications could not only affect its DNA and/or protein binding properties, but also its biophysical behavior. SATB1 does not possess an IDR and serine bias as strong as typical phase separating proteins (Figure 4). However, SATB1 could function similarly to the signaling molecules like STAT3, SMAD3 and β-catenin which use their IDRs (Figure 4) to target phase separated transcriptional and/or other condensates [116]. One IDR region of SATB1 is directly extended by a poly-Q region, which is also known to drive phase separation [104,105,117,118].

Although, the liquid-liquid phase separation model is currently very popular in the literature, there are alternative models which may provide more accurate description of the biophysical behavior of certain proteins [119–121]. Thus, we would like not to draw any conclusion from our observations about SATB1 and phase separation, we would rather like to emphasize the lack of biophysical studies on SATB1. SATB1 is unequivocally a versatile and extremely interesting protein which would deserve more attention from the biophysical perspective, similar to 21. The new studies should optimally be focused on individual SATB1 variants, e.g. utilizing specific mutations affecting post-translational modifications and/or different protein isoforms and, where possible, all this should be investigated with respect to the primary T cell developmental stages.

### SATB1 as a repressor, activator or just a scaffold

Multiple studies of SATB1 have indicated its variegated nature depending on the cellular context. The ambiguity of SATB1 in terms of its functions started with the original studies calling SATB1 a potent repressor [122–124]. The suppressive role of SATB1 was further supported by other studies, e.g. SATB1 can directly repress the *Il5* gene in Th2 cells [46] and/or *Myc, Numb* and possibly other genes in hematopoietic stem cells [37]. Another set of studies demonstrated that SATB1 has also a positive impact on gene expression. It creates activatory chromatin loops at the Th2 locus promoting the expression of Th2 signature genes [80]. In general, it seems that most of its activatory potential resides at the DP stage where SATB1 is also predominantly expressed [37,39–41]. In DP thymocytes, SATB1 mediates activatory loops and it was found on enhancers regulating *Rag1* and *Rag2* genes [91], Treg signature genes [39] as well as genes encoding master regulators ThPOK and *Runx3* [50]. Moreover, it promotes expression of co-receptor genes *Cd4* [50] and *Cd8* [50,125]. The connection between regulation of the key T cell genes and high levels of SATB1 at the DP stage could fit with our hypothetical model that the sudden increase in SATB1 levels at the DP stage triggers its phase separation into transcriptional condensates, while bringing together the T cell specific genes and their regulatory elements where SATB1 is bound.

However, both positive and negative functions may also be executed solely via the recruitment of chromatin modifying complexes [5,9,45,78,126–129]. The question remains how would be the entire system regulated to decide which function prevails at the moment. A suggested model operates with the post-translational modifications of SATB1, serving as a molecular switch [45,129]. Many more post-translational modifications are predicted together with two SATB1 protein isoforms (Figure 1), altogether generating a large pool of potential SATB1 variants. Both, the cellular context, especially the developmental stage, and the number of SATB1 variants potentially available, represent a crucial variable that should be considered in genome-wide studies employing the commercially available anti-SATB1 antibodies which cannot discriminate between any of the aforementioned variants.

**Figure 5.**
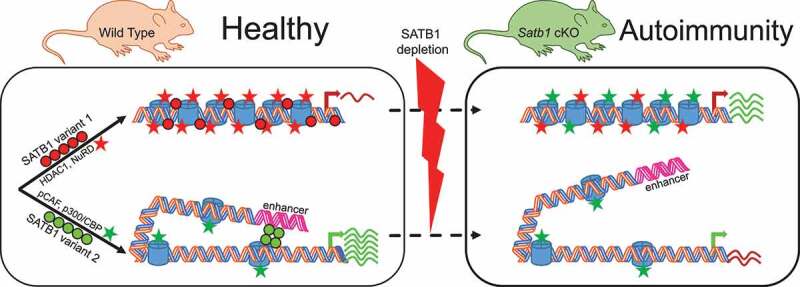
Proposed model on SATB1’s diverse modes of action and how their deregulation may result to disease. Different variants of SATB1 introduced in Figures 1 and 3 can define quite diverse interactomes localized within the cell nucleus. Consequently, different chromatin modifying or remodeling complexes are recruited by different SATB1 variants to the regulated genes. Chromatin accessibility is modified and transcription activation or repression occurs. Moreover, SATB1 mediates long-range promoter-enhancer communication and ultimately regulates chromatin organization. In its absence, the interactome and/or the loopscape structure of the genome is altered resulting in a modified transcriptome. Here we demonstrate how transcription of two genes is controlled either positively or negatively by two distinct SATB1 variants. This transcription state is deregulated in the *Satb1* conditional knockout mice, ultimately leading to a disease such as autoimmunity. A link between deregulated SATB1 protein and altered chromatin landscape and/or genome organization in human autoimmune diseases has not been thoroughly studied yet.

Overall, we propose that there are several functional SATB1 variants present in the nucleus, each exerting a slightly different role (Figure 5). It is possible that some variants tend to create a scaffold in line with the model of nuclear matrix. Binding of some of these variants may repress certain loci by competing with activatory factors of gene expression, by directly attracting other repressive complexes or by other mechanisms. The scaffold variants may prepare a poised, T cell specific, chromatin conformation. Upon post-translational modification, the new SATB1 variants may mediate chromatin looping to transcriptionally active nuclear zones, while mediating enhancer-promoter communication and serving as a docking station for activatory chromatin modifying complexes (Figure 3).

## Importance in human physiology

In the absence of SATB1, specifically in T cells, mice develop an autoimmune-like phenotype accompanied by inflammation. These mice display a dysfunction for the positive selection process in the thymus thus leading to a blockade in transition from the DP to the CD4SP or CD8SP cell stage [49]. Mice die prematurely, with observed increased concentration of autoantibodies in the serum, infiltration of effector T cells in various tissues and an impairment of regulatory T cells [39,40]. Therefore, ablation of SATB1 has a major effect on immune tolerance in mice. Whether such a mechanism exists in humans remains to be elucidated, which would render SATB1 protein as a prognostic marker for early detection of autoimmune diseases.

SATB1 has also been under investigation in the cancer research field due to its high expression in several malignancies [130–132], supporting its action as a tumor promoter. SATB1 may have an important role as a positive regulator of glioma development and progression and it may serve as a useful molecular marker for predicting its prognosis [133]. It is important in the initiation of colorectal cancer and SATB1 expression in colorectal cancer is associated with the expression of S100A4 [134], MMP2, NF-kB, PCNA [135], cyclin D1 and β-catenin [136,137]. Therefore, a lot of reports support the idea that SATB1 expression is a biomarker predicting the poor prognosis in colorectal cancer patients [10,138]. Moreover, it is the high expression level of SATB1 that relates to poor prognosis in colorectal cancer patients [136,139]. Analogically to CTCF, a potential mode of action for SATB1 as a genome organizer, is the establishment of cell-specific long-range interactions (Figures 3 and 5). In that scenario, the alteration in chromatin structure, either due to changes in protein expression and/or DNA binding properties or due to DNA polymorphisms causing different structural variants could potentially all lead to a malignant output [140].

## Conclusions

SATB1 is a multivalent protein building a putative nuclear scaffold, recruiting chromatin modifying enzymes, mediating long-range chromatin interactions and generally participating in expression regulation of many genes at distinct levels. It is becoming clear that all these functions and often the bi-potential regulatory role are exerted by multiple forms of SATB1. Future research of the two protein isoforms as well as copious post-translational modifications multiplying the number of functional SATB1 variants could provide at least some answers. Although, there are several great studies focused on its roles during the intrathymic T cell development, thorough genome-wide studies with respect to its chromatin organization abilities are still missing. SATB1 has a potential to complement the set of traditional lineage specifying factors at the double positive T cell stage and maybe even earlier in the development. Chromatin organization mediated by SATB1 is unequivocally of high importance for the expression regulation of several hand-picked loci studied. However, a top-down search for all the transcriptional programs affected by the lack of SATB1 is still needed. Post-translational modifications studied at the N-terminus of SATB1 influence its protein-protein interaction properties. However, the C-terminus of SATB1 also harbors several predicted sites for phosphorylation, ubiquitination and possibly other modifications. It would be interesting to see how these modifications change the DNA binding properties of SATB1 and the overall SATB1-mediated 3D architecture of the T cell genome (Figures 3 and 5). The technological advances allow us to revisit nuclear localization of SATB1 at nanometer scale together with its dynamics and other biophysical qualities. It is becoming clear that there is a link between 3D chromatin organization, phase separation and regulation of basic biological processes such as transcription, replication and others. Therefore, the SATB1 research should adapt toward more interdisciplinary approaches. More so, as there exist multiple SATB1 variants with most likely very different molecular functions. Moreover, the cellular context is extremely important and (not only) in the case of SATB1 it is closely related to its function. Thus, the lack of focus on a specific developmental stage of primary cells is another limitation of current studies. Research of primary thymocytes is constrained by multiple technical limitations, however as the technology advances, it is time to revisit some old concepts. Last but not least, the increasing demand for basic SATB1 research in clinical studies is the ultimate evidence that there is enough room for new discoveries.
